# Retraction: Biodegradation of the Allelopathic Chemical *m*-Tyrosine by *Bacillus aquimaris* SSC5 Involves the Homogentisate Central Pathway

**DOI:** 10.1371/journal.pone.0102854

**Published:** 2014-07-09

**Authors:** The PLOS ONE Editors

The Council of Scientific and Industrial Research has carried out an investigation about several publications by this group in order to evaluate concerns raised about the authenticity of the data.

The investigation committee at the Council of Scientific and Industrial Research has concluded that there are no data available underlying this study and thus that the published results are fabricated. As a result, the Council of Scientific and Industrial Research has requested the retraction of the publication. The authors are in agreement with the request by the investigation committee.

In line with the outcome of the institutional investigation, *PLOS ONE* retracts this publication.

## References

[1] Khan F, Kumari M, Cameotra SS (2013) Biodegradation of the Allelopathic Chemical *m*-Tyrosine by *Bacillus aquimaris* SSC5 Involves the Homogentisate Central Pathway. PLoS ONE 8(10): e75928 doi:10.1371/journal.pone.0075928 24098407 10.1371/journal.pone.0075928 PMC3788032

